# Metagenomic prediction of antimicrobial resistance in critically ill patients with lower respiratory tract infections

**DOI:** 10.1186/s13073-022-01072-4

**Published:** 2022-07-12

**Authors:** Paula Hayakawa Serpa, Xianding Deng, Mazin Abdelghany, Emily Crawford, Katherine Malcolm, Saharai Caldera, Monica Fung, Aaron McGeever, Katrina L. Kalantar, Amy Lyden, Rajani Ghale, Thomas Deiss, Norma Neff, Steven A. Miller, Sarah B. Doernberg, Charles Y. Chiu, Joseph L. DeRisi, Carolyn S. Calfee, Charles R. Langelier

**Affiliations:** 1 Division of Infectious Diseases, Department of Medicine, University of California, San Francisco, San Francisco, CA USA; 2 Chan Zuckerberg Biohub, San Francisco, CA USA; 3 Department of Laboratory Medicine, University of California, San Francisco, CA USA; 4 Department of Microbiology and Immunology, University of California, San Francisco, CA USA; 5 Division of Pulmonary, Critical Care, Allergy and Sleep Medicine, Department of Medicine, University of California, San Francisco, CA USA; 6 Chan Zuckerberg Initiative, San Francisco, CA USA; 7 Department of Biochemistry and Biophysics, University of California, San Francisco, CA USA

## Abstract

**Background:**

Antimicrobial resistance (AMR) is rising at an alarming rate and complicating the management of infectious diseases including lower respiratory tract infections (LRTI). Metagenomic next-generation sequencing (mNGS) is a recently established method for culture-independent LRTI diagnosis, but its utility for predicting AMR has remained unclear. We aimed to assess the performance of mNGS for AMR prediction in bacterial LRTI and demonstrate proof of concept for epidemiological AMR surveillance and rapid AMR gene detection using Cas9 enrichment and nanopore sequencing.

**Methods:**

We studied 88 patients with acute respiratory failure between 07/2013 and 9/2018, enrolled through a previous observational study of LRTI. Inclusion criteria were age ≥ 18, need for mechanical ventilation, and respiratory specimen collection within 72 h of intubation. Exclusion criteria were decline of study participation, unclear LRTI status, or no matched RNA and DNA mNGS data from a respiratory specimen. Patients with LRTI were identified by clinical adjudication. mNGS was performed on lower respiratory tract specimens. The primary outcome was mNGS performance for predicting phenotypic antimicrobial susceptibility and was assessed in patients with LRTI from culture-confirmed bacterial pathogens with clinical antimicrobial susceptibility testing (*n* = 27 patients, *n* = 32 pathogens). Secondary outcomes included the association between hospital exposure and AMR gene burden in the respiratory microbiome (*n* = 88 patients), and AMR gene detection using Cas9 targeted enrichment and nanopore sequencing (*n* = 10 patients).

**Results:**

Compared to clinical antimicrobial susceptibility testing, the performance of respiratory mNGS for predicting AMR varied by pathogen, antimicrobial, and nucleic acid type sequenced. For gram-positive bacteria, a combination of RNA + DNA mNGS achieved a sensitivity of 70% (95% confidence interval (CI) 47–87%) and specificity of 95% (CI 85–99%). For gram-negative bacteria, sensitivity was 100% (CI 87–100%) and specificity 64% (CI 48–78%). Patients with hospital-onset LRTI had a greater AMR gene burden in their respiratory microbiome versus those with community-onset LRTI (*p* = 0.00030), or those without LRTI (*p* = 0.0024). We found that Cas9 targeted sequencing could enrich for low abundance AMR genes by > 2500-fold and enabled their rapid detection using a nanopore platform.

**Conclusions:**

mNGS has utility for the detection and surveillance of resistant bacterial LRTI pathogens.

**Supplementary Information:**

The online version contains supplementary material available at 10.1186/s13073-022-01072-4.

## Background

Antimicrobial resistance (AMR) presents a clear threat to human health and is responsible for increasing rates of treatment failure in patients with lower respiratory tract infections (LRTI), the leading cause of infectious disease-related mortality [1]. Implementing effective and targeted therapies in patients with LRTI necessitates not only accurate detection of a broad range of pathogens, but also requires assessment of their resistance to antimicrobials. In many cases, assessment of AMR is not possible due to the need to first isolate a bacterial pathogen in culture prior to antimicrobial susceptibility testing (AST), a process that can require several days and have low yield in the setting of prior antibiotic use [2, 3]. In the absence of a definitive microbiologic diagnosis, LRTI treatment is by necessity empiric, which leads to broad-spectrum antibiotic overuse and selects for resistant pathogens [4, 5].

Metagenomic next-generation sequencing (mNGS) holds promise for overcoming the limitations of traditional respiratory diagnostics by affording culture-independent detection of pathogens and simultaneous profiling of host gene expression signatures of infection [6]. In principle, mNGS can also be used to predict pathogen AMR by detecting bacterial resistance genes. While the performance of cultured bacterial isolate whole genome sequencing has been extensively characterized [7], studies assessing the performance of direct respiratory specimen mNGS for predicting AMR have remained more limited [8–12].

This is in part due to the low abundance of pathogen AMR genes in respiratory and other clinical body fluids, which challenges their detection using conventional mNGS methods [12]. Recent work has demonstrated the potential for CRISPR/Cas9 targeted enrichment using FLASH (Finding Low Abundance Sequences by Hybridization) to overcome this challenge by enhancing detection of low abundance AMR genes in clinical samples. Independent validation of FLASH in a clinical cohort, however, has been needed.

Here, we address these gaps by studying a cohort of critically ill patients to assess the potential of both DNA and RNA mNGS to predict LRTI bacterial pathogen AMR, facilitate epidemiological AMR surveillance, and rapidly detect clinically relevant resistance genes using CRISPR/Cas9 targeted enrichment coupled with real-time nanopore sequencing.

## Methods

### Study design

**Fig. 1:**
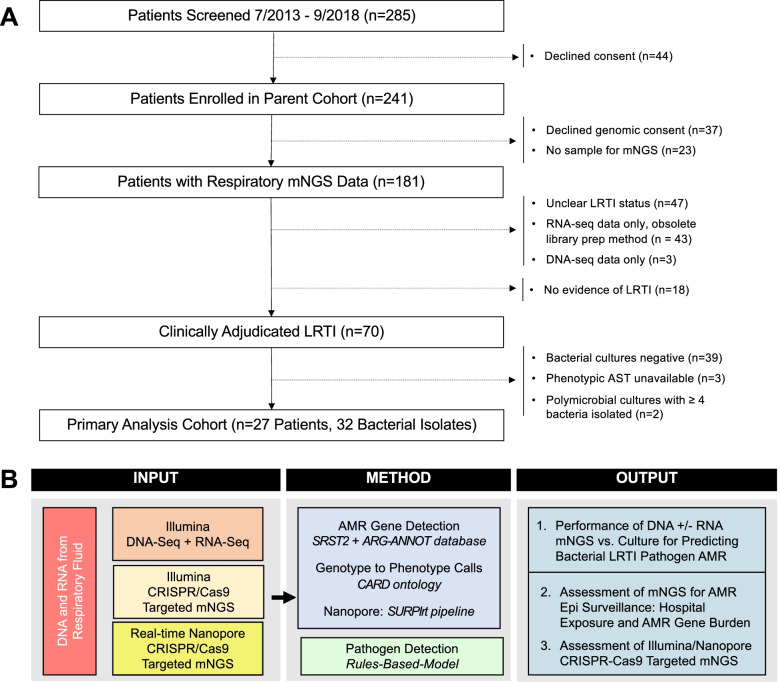
Study overview and analysis workflow.** A** Enrollment flow diagram for the critically ill adult cohort with acute respiratory illnesses that was studied. **B** Metagenomic next-generation sequencing (mNGS) approach and analysis workflow. The primary analysis assessed the performance of metagenomic next-generation sequencing (mNGS) antimicrobial resistance (AMR) prediction in 27 subjects with LRTI due to 32 culture-confirmed bacterial pathogens. Secondary analyses included mNGS epidemiological assessment of hospital exposure and AMR gene burden in the airway microbiome, and proof of concept assessment of CRISPR/Cas9 targeted mNGS using Illumina and real-time nanopore sequencing

We studied 70 mechanically ventilated patients with LRTI and 18 with non-infectious respiratory illnesses (Fig. 1, Additional File 1: Table S1) who were admitted to the University of California San Francisco (UCSF) Medical Center between 07/2013 and 9/2018. Subjects with LRTI were identified by two-physician adjudication using the United States Centers for Disease Control/National Healthcare Safety Network (CDC/NHSN) surveillance case definition [13], a reference list of established respiratory pathogens [6], and retrospective electronic medical record review, blinded to mNGS results. Study inclusion criteria were age ≥ 18, need for mechanical ventilation, and lower respiratory specimen (tracheal aspirate (TA) or mini-bronchoalveolar lavage (mBAL)) collected within 72 h of intubation. Patients were excluded if they declined study participation, had unclear LRTI status, or did not have matched RNA and DNA mNGS data available from a respiratory specimen (Fig. 1).

Primary analyses were performed for 27 patients, secondary analyses for all subjects. The primary analysis focused on patients with bacterial LRTI due to culture-confirmed pathogens that had been clinically tested for susceptibility to antimicrobials (*n* = 27 patients, *n* = 32 pathogens) (Fig. 1, Table 1, Additional File 2: Table S2). Of these, 18 patients had respiratory samples sequenced for a prior mNGS study by our group [6]. For secondary analyses, 43 additional patients with clinically adjudicated LRTI and 18 patients with no evidence of LRTI were assessed. In total, the secondary outcome analysis of hospital exposure and AMR gene burden in the respiratory microbiome assessed 70 patients with LRTI and 18 patients with no evidence of LRTI. Assessment of Cas9 targeted Illumina and nanopore sequencing for detecting AMR genes included 10 patients from the primary analysis with culture-confirmed bacterial LRTI.

**Table 1:**
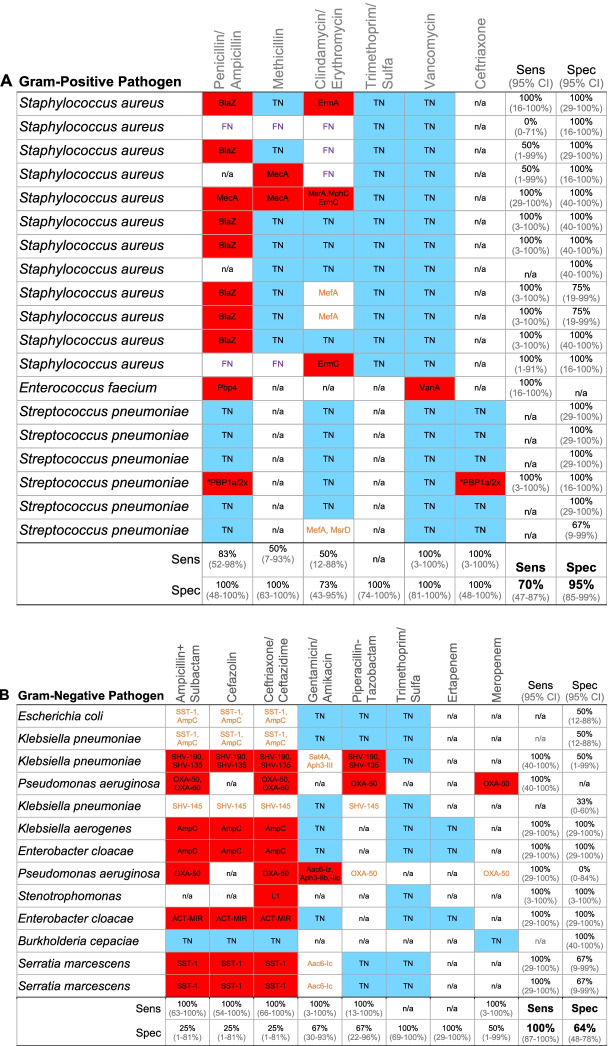
Performance of mNGS for genotypic prediction of antimicrobial susceptibility compared to a reference standard of clinical microbiologic testing. Sensitivity, specificity, and accuracy of DNA + RNA mNGS compared to a reference standard of clinical antimicrobial susceptibility testing based on Clinical & Laboratory Standards Institute (CLSI) minimum inhibitory concentration (MIC) breakpoints*.* A Gram-positive pathogens. B Gram-negative pathogens. AMR gene(s) detected by mNGS indicated. With respect to genotype-phenotype predictions, squares filled red indicate true positives, squares filled blue indicate true-negatives, squares with purple text = false negatives, squares with orange text are false positives

*mutations in PBP1a/2x, *Sens* Sensitivity, *Spec* Specificity, *TN* True negative, *FN* False negative; *n/a* phenotypic susceptibility to antibiotic not tested in the clinical laboratory. 95% confidence interval (CI) listed below each sensitivity and specificity value

### Procedures

#### Nucleic acid extraction and Illumina metagenomic sequencing

RNA extraction from mBAL or TA and Illumina metagenomic sequencing were carried out as described previously [6, 14].

#### Pathogen detection bioinformatics

Detection of respiratory microbes leveraged the ID-Seq pipeline [14] that incorporates the STAR [15] aligner to subtract the human genome (NCBI GRC h38), quality filtering with PRICESeqfilter [16], and additional filtering to remove non-microbial sequences. The identities of the remaining microbial reads were determined by querying the NCBI nucleotide (NT) and non-redundant protein (NR) databases using GSNAP-L and RAPSEARCH2, respectively [14]. Microbial alignments detected by RNA-seq and DNA-seq were aggregated to the genus level and the sequencing reads comprising each genus were then evaluated for taxonomic assignment at the species level based on species relative abundance. A recently developed rules-based model (RBM) [6] was employed to differentiate putative pathogens from commensal microbiota.

The RBM leverages previous findings demonstrating that microbial communities in patients with LRTI are typically characterized by one or more dominant pathogens present in high abundance [6, 14]. More specifically, the RBM ranks microbial genera present in a sample by descending abundance (number of taxonomic alignments). The greatest difference between any two sequential taxa is then identified to capture genera present at disproportionately high abundance compared to the rest of the lung microbiota [6, 17].

All genera with an abundance greater than this largest gap threshold are then evaluated at the species level, by identifying the most abundant species within each genus. If the species is present within a previously curated reference index of established respiratory pathogens [6, 17] derived from landmark epidemiologic surveillance studies [18–22], it is selected as a putative pathogen by the RBM. A detailed description of the principles and clinical validation of the RBM has been previously published [6, 17].

#### Detection of AMR genes

AMR genes present in RNA-seq or DNA-seq data were identified using SRST2 coupled with an expanded version of the ARG-ANNOT database [23] (Additional File 3), and genes with ≥ 5% allele coverage were included in analyses. Because *Streptococcus pneumoniae* is a leading cause of bacterial LRTI [4], we also screened for point mutations in *pbp* genes associated with *Streptococcus* beta lactam resistance using the CARD resistance gene identifier tool and the ‘loose’ setting [24]. Average read depth across each allele, normalized by gene length and total reads (depth per million reads sequenced, dpM), was calculated for each sample.

#### Assessing performance of genotypic antimicrobial susceptibility prediction

As a reference standard, we used clinical AST results performed in the UCSF Clinical Microbiology Laboratory during each patient’s admission. To calculate sensitivity and specificity, which was done both by microbe and by drug (Table 1), we compared mNGS-based resistance predictions against phenotypic AST determined by the Clinical & Laboratory Standards Institute minimum inhibitory concentration breakpoints [25]. We studied samples from subjects with culture-confirmed bacterial pathogens for which AST was performed. One isolate that only underwent chromogenic beta lactamase screening was excluded. Two subjects (252, 297) with highly polymicrobial cultures of ≥ 4 organisms were also excluded due to the unclear clinical significance of the isolated microbes. This left a primary analysis cohort of 27 patients and 32 bacterial pathogens (Fig. 1).

We assessed susceptibility to the most common antibiotics used for complicated infections from bacterial pathogens identified in the cohort: *S. aureus, S. pneumoniae, E. faecium*, *Enterobacteriaceae, P. aeruginosa*, and *S. maltophila*. Initial AMR gene class assignment (beta lactam, aminoglycoside, macrolide/lincosamide/ streptogramin, glycopeptide, trimethoprim/ sulfamethoxazole) was made using ontology in ARG-ANNOT [23] and a more refined AMR phenotype assignment was made based on CARD [24] resistome ontological relationships. In addition to sensitivity and specificity, we assessed very major error (VME; predicted susceptible but phenotypically resistant) and major error (ME; predicted resistant but phenotypically susceptible) rates.

#### Clinically tested antimicrobials used in mNGS AMR prediction benchmarking

Resistance predictions were made for antibiotics routinely tested in the clinically microbiology laboratory for *Staphylococcus aureus, Streptococcus pneumoniae, Enterococcus faecium*, *Pseudomonas aeruginosa*, *Stenotrophomonas maltophila,* and *Enterobacteriaceae*. For *S. aureus* these included penicillin, methicillin, clindamycin or erythromycin, trimethoprim/ sulfamethoxazole (TMP/SMZ), and vancomycin; for *S. pneumoniae*: penicillin, ceftriaxone, and vancomycin; for *E. faecium:* ampicillin and vancomycin; for *Enterobacteriaceae*: ampicillin + sulbactam, cefazolin, ceftriaxone, gentamicin, piperacillin-tazobactam, TMP-SMX, ertapenem, and meropenem; for *P. aeruginosa:* ampicillin + sulbactam, ceftazidime, gentamicin, piperacillin-tazobactam, and meropenem; and for *S. maltophila:* ceftazidime and TMP/SMZ. For some isolates, clinical susceptibility testing for certain antimicrobials was not performed by the clinical laboratory, and thus was unavailable for our analysis.

#### FLASH Cas9 targeted mNGS for AMR gene detection

FLASH Cas9 targeted Illumina mNGS for AMR gene detection was carried out as described in the original proof of concept study [12]. Briefly, FLASHit software [26] was first used to design guide RNAs targeting clinically relevant AMR genes derived from the CARD and ResFinder databases, merging exact duplicates [12]. In total, 2226 guide RNAs targeting 381 beta lactam and 111 MLS resistance genes, in addition to the 127 diverse AMR genes from the original FLASH pilot study, were utilized for Cas9 targeted enrichment. Guide RNAs targeted multiple sites on each AMR gene, which in total represented 2226 target sequences (Additional File 4). DNA templates for producing CRISPR RNAs (crRNAs) for each AMR gene target were synthesized, pooled, transcribed, and purified according to described methods [12].

Ten nanograms of DNA was 5′ dephosphorylated using rAPid alkaline phosphatase that was subsequently deactivated with sodium orthovanadate. The dephosphorylated DNA was added to a master mix containing the CRISPR/Cas9 ribonucleoprotein complex and incubated at 37 °C for 2 h. The Cas9 was deactivated with proteinase K and removed with SPRI bead purification. Samples were dA-tailed and then carried forward for Illumina Sequencing according to the NEBNext Ultra II library prep kit (New England Biolabs, Ipswich, MA) protocol according to previously described detailed methods [12]. AMR gene identification was carried out using ARG-ANNOT [23] as for the primary analyses, and genes that were detected at a dpM of > 0.1 were assessed for enrichment compared to DNA-seq alone.

#### Nanopore sequencing

FLASH-enriched DNA libraries were quantified and 200–800 ng of DNA input was used for Nanopore 1D library preparation (protocol SQK-LSK109, Oxford Nanopore, UK). Individual sample libraries were loaded into a single flow cell of a GridION instrument, and sequencing reads were base called in real-time mode in MINKNOW. The SURPIrt pipeline running in -a mode was utilized to identify AMR genes every 100,000–200,000 reads as previously described [27, 28].

#### Mitigation of background contaminants

To minimize inaccurate taxonomic assignments due to environmental contaminants, we processed negative water controls with each group of samples that underwent nucleic acid extraction, and included these, as well as positive control clinical samples, with each sequencing run. We directly subtracted alignments to those taxa in water control samples detected by both RNA-seq and DNA-seq analyses from the raw reads per million (rpm) values in all samples [6]. To account for selective amplification bias of contaminants in water controls resulting from PCR amplification of metagenomic libraries to a fixed standard concentration across all samples, prior to direct subtraction, we scaled taxa rpms in the water controls to the median percent microbial reads present across all samples as previously described [6]. To address environmental contaminants in AMR gene analyses, resistance alleles detected in water controls at a depth > 1 were excluded.

### Study outcomes

The primary outcome was performance of mNGS for predicting phenotypic AST. Secondary outcomes included the association between hospital exposure and burden of AMR genes in the respiratory microbiome and AMR gene detection using Cas9 targeted enrichment and real-time nanopore sequencing.

### Statistical analysis

Statistical significance was defined as *P* less than 0.05, using two-tailed tests of hypotheses. Nonparametric continuous variables were analyzed by Wilcoxon rank-sum.

## Results

### Cohort features

Seventy subjects with LRTI and 18 with no evidence of LRTI were identified based on inclusion and exclusion criteria (Fig. 1, Additional File 1: Table S1). Primary analyses were performed for 27 patients, secondary analyses for all subjects. Clinical AST results were returned a median of 74 h following sample collection (95% confidence interval (CI) 49–115 h, (Additional File 5: Table S3)). Twenty-seven subjects with culture-confirmed bacterial LRTI, representing 32 pathogens with clinical AST data performed on ≥ 2 drugs, were identified and assessed in the primary analysis (Additional File 2: Table S2). For secondary analyses, 43 additional patients with clinically adjudicated LRTI and 18 patients with no evidence of LRTI were assessed.

### Metagenomic sequencing, pathogen, and AMR gene detection

A mean of 4.3 × 10^7^ (interquartile range (IQR) 1.9–4.4 × 10^7^) DNA-seq reads and 6.9 × 10^7^ (IQR 4.8–8.3 × 10^7^) RNA-seq reads were generated from respiratory samples. In the primary AMR analysis group, we used a previously validated [6] metagenomic rules-based model (RBM) to identify bacterial respiratory pathogens that were disproportionately abundant as compared to the rest of the lung microbiome. The RBM identified 26 of 32 (81%) of the culture-confirmed bacterial pathogens from the primary analysis. Four (67%) of the missed pathogens were present in the context of polymicrobial cultures, and one (17%) was identified as a different streptococcal species (Additional File 2: Table S2). A total of 138 and 234 acquired AMR genes were identified by RNA-seq and DNA-seq, respectively (Additional File 6: Table S4). With respect to AMR gene classes, beta lactam resistance genes were most common (81/372 total genes, 35%).

### Comparison of mNGS versus phenotypic antimicrobial susceptibility testing

We assessed the performance of mNGS for predicting resistance to clinical guideline-recommended antimicrobials used for complicated gram-negative (*n* = 8 drugs) and gram-positive (*n* = 6 drugs) infections. AMR genes unrelated to the culture-confirmed bacterial pathogen were identified through the resistome ontology annotations in CARD [24] and excluded from this analysis. Sensitivity and specificity compared to a reference standard of culture-based AST varied by pathogen, drug, patient, and nucleic acid type sequenced (Table 1, Additional File 7: Table S5). For gram-positive pathogens, a combination of DNA-seq and RNA-seq yielded a sensitivity of 70% (CI 47–87%), specificity of 95% (CI 85–99%), and an accuracy of 87% (CI 78–94%) (Table 1). This equated to a VME rate of 30% and a ME rate of 5%. For gram-negative pathogens, a combination of DNA-seq and RNA-seq yielded a sensitivity of 100% (CI 87–100%), specificity of 64% (CI 48–78%), and accuracy of 78% (CI 67–87%) (Table 1). This equated to a VME rate of 0% and a ME rate of 36%.

We also assessed the performance of RNA-seq and DNA-seq performed independently (Table 1, Additional File 7: Table S5). RNA-seq performed with a sensitivity of 52% (CI 31–73%), specificity of 100% (CI 94–100%), and accuracy of 86% (CI 76–93%) for gram-positive pathogens, and a sensitivity of 100% (CI 89–100%), specificity of 64% (CI 48–78%), and accuracy of 79% (CI 68–88%) for gram-negative pathogens. DNA-seq performed with a sensitivity of 39% (CI 20–61%), specificity of 95% (CI 85–99%), and accuracy of 78% (CI 67–87%) for gram-positive pathogens, and a sensitivity of 58% (CI 39–75%), specificity of 67% (CI 50–80%), and accuracy of 63% (CI 51–74%) for gram-negative pathogens.

In two of seven cases with genotype to phenotype false-positive (ME) predictions, mNGS identified AMR genes unrelated to the culture-confirmed microbe but related to resistant pathogens that would be cultured several days later in the context of ventilator-associated pneumonia (VAP). These included *SST-1* from a patient who developed *Serratia marcescens* VAP (patient 213) 4 days later, and *mecA* from a patient who developed *Staphylococcus aureus* VAP 7 days later (patient 232) (Additional File 6: Table S4).

### Association between LRTI positivity, hospital exposure, and AMR genes in the respiratory microbiome

**Fig. 2:**
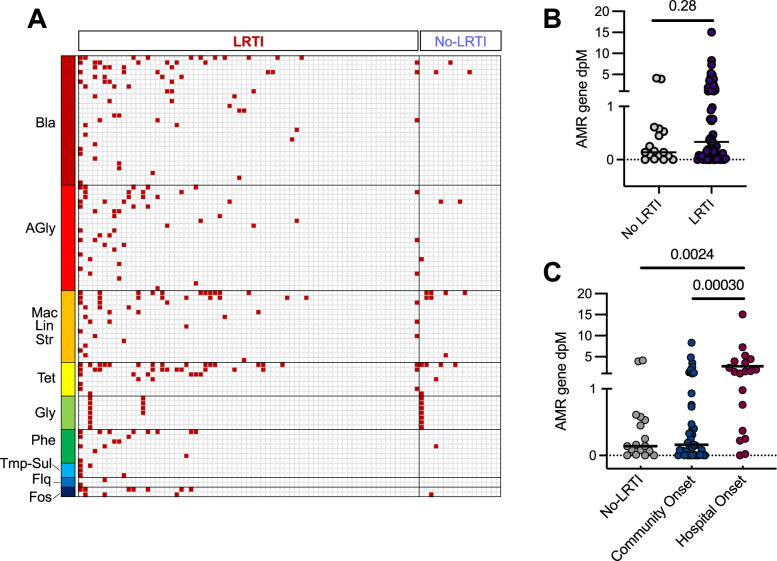
**A** AMR genes detected in the lower respiratory microbiome of critically ill patients. Composite results of DNA and RNA mNGS. AMR genes are listed in rows and are grouped by antimicrobial class. Each column represents a patient respiratory sample and is grouped by LRTI status. **B** AMR gene burden in the respiratory tract, measured by averaging sequencing depth across the AMR allele per million reads sequenced (dpM) in the respiratory microbiome did not differ between LRTI-positive patients and those with non-infectious acute respiratory illnesses. **C** The burden of AMR genes detected in the lower respiratory tract microbiome was greater in patients with hospital-onset LRTI versus those with either community-onset LRTI or no evidence of LRTI. Legend: depth = average sequencing depth across each AMR gene allele normalized per million reads sequenced. *Legend*: Bla = beta lactam; AGly = aminoglycoside; Fos = Fosfomycin; Flq = fluoroquinolone; Gly = glycopeptide; Mac/Lin/Str = macrolide, lincosamide, streptogramin; Phe = phenicol; Tet = tetracycline; Tmp-Sul = trimethoprim/sulfamethoxazole; depth = average sequencing depth across each AMR gene allele normalized per million reads sequenced. The horizontal bars in panels **B** and **C** indicate mean values

Assessment of the lower respiratory resistome using both DNA and RNA mNGS revealed a diversity of AMR genes in both LRTI-positive and negative patients (Fig. 2A, Additional File 6: Table S4). AMR gene burden did not differ based on LRTI status (*p* = 0.28) (Fig. 2B). Subjects with hospital-onset (≥ 48 h after admission) LRTI had a greater burden of AMR genes in their respiratory microbiome compared to those with community-onset LRTI (*p* = 0.00030) or those without LRTI (*p* = 0.0024) (Fig. 2C).

### Targeted enrichment and rapid detection of AMR genes using Cas9 and nanopore sequencing

**Fig. 3:**
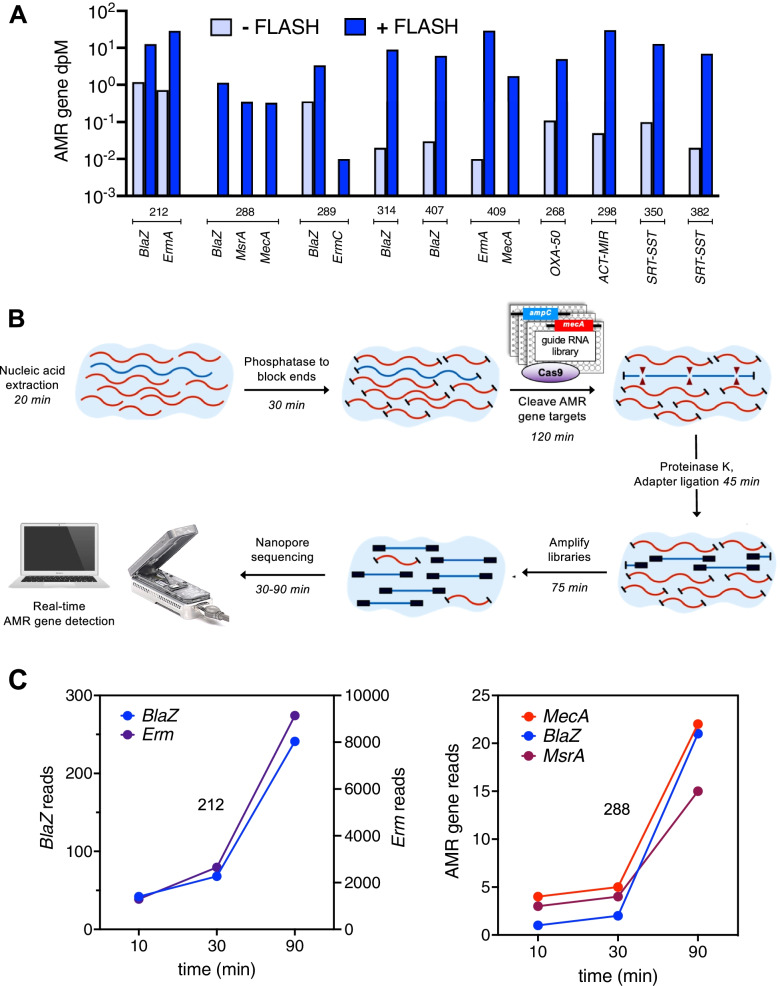
**A** FLASH (Finding Low Abundance Sequences by Hybridization) CRISPR/Cas9 targeted Illumina sequencing enriched the detection of culture-confirmed bacterial LRTI pathogen AMR alleles by 46 × to > 2500 × versus DNA-seq alone. **B** Workflow diagram for FLASH targeted enrichment coupled with nanopore sequencing. Time estimates provided for a single sample. **C** Real-time detection of AMR genes by FLASH targeted nanopore sequencing was achieved within 10 min following mNGS library preparation. Data from two representative *Staphylococcus aureus* LRTI cases are highlighted. Case 212 (left panel) highlights a case where detection of *BlaZ* and *MsrA/ErmA* genes correlated with phenotypically determined penicillin and macrolide/lincosamide resistance, respectively. Case 288 (right panel) highlights a case where detection of *MecA, BlaZ, and MsrA* correlated with phenotypically confirmed methicillin, penicillin, and macrolide resistance, respectively

We validated the utility of a recently described programmable CRISPR/Cas9-based method called FLASH (Finding Low Abundance Sequences by Hybridization) to enrich for low abundance AMR genes [12] by studying 10 patients from the primary analysis (Additional File 8: Table S6). The FLASH + DNA mNGS library prep protocol added 2.5 h to the standard 3-h NEBNext DNA-seq workflow [29] and could enrich detection of AMR genes associated with the culture-confirmed pathogen > 2500 × compared to DNA-seq alone (Fig. 3A). In four (40%) patients, FLASH enabled detection of AMR genes that were associated with the culture-confirmed pathogen and resistance phenotype, but missed by DNA-seq alone, including *mecA* in two patients with methicillin-resistant *Staphylococcus aureus* LRTI. FLASH also resulted in the detection of AMR genes unrelated to the culture-confirmed pathogens in five (50%) of patients (Additional File 8: Table S6).

We subsequently assessed the potential for rapid AMR gene detection using FLASH combined with an Oxford nanopore sequencing platform, which affords real-time data generation (Fig. 3B). All AMR genes identified by Illumina (median sequencing depth of 1.20 × 10^8^ [IQR 5.41 × 10^7^–1.36 × 10^8^]) were also identified by FLASH-nanopore sequencing (median sequencing depth of 1.19 × 10^6^ reads [IQR 1.02 × 10^6^–1.46 × 10^6^]) (Additional File 9: Table S7). AMR gene targets could be identified within 10 min of real-time nanopore sequencing, suggesting a potential turnaround time of less than 6 h for a single sample (Fig. 3C).

## Discussion

Antimicrobial resistance has emerged as one of the most pressing issues facing human health, and effective treatment of complicated infections increasingly necessitates early and accurate assessment of microbial drug resistance. Our study builds on prior respiratory mNGS studies [6, 8–12] by demonstrating that RNA and DNA mNGS can enable culture-independent prediction of AMR in critically ill patients with bacterial LRTI, with variable performance across pathogens and antimicrobials. While false-negative susceptibility predictions for gram-negative pathogens were not observed, we found a VME of 30% for gram-positive pathogens, suggesting mNGS has a role for complementing, rather than replacing, current standard of care culture-based approaches.

Prior work has demonstrated the utility of mNGS in cases of culture-negative LRTI, which represent more than half of all pneumonia cases [4, 6, 8, 10]. Our results suggest that mNGS may also have potential for predicting AST in culture-negative LRTI, where detection of an AMR gene could inform the need for treatment of an occult resistant organism. Further, our findings support recent observations [10] that mNGS may have utility for early identification of future secondary respiratory infections. For instance, patient 232, who was admitted for severe *Klebsiella pneumoniae* LRTI, developed MRSA ventilator-associated pneumonia (VAP) eight days later while undergoing treatment with aztreonam, an antibiotic lacking MRSA coverage. mNGS analysis of a TA specimen obtained 7 days before VAP onset revealed the *mecA* gene, providing an early indication of the subsequent AMR VAP pathogen.

Public health surveillance is essential for pandemic preparedness, understanding trends in AMR, and preventing outbreaks of resistant organisms. Our findings demonstrate the feasibility of mNGS for epidemiological surveillance of AMR and highlight an association between hospital exposure and AMR gene burden in the lower respiratory tract microbiome. Importantly, our analysis included culture-negative LRTI cases and adds to prior literature demonstrating a similar association in cases of culture-confirmed bacterial pneumonia [30].

Nanopore sequencing has proven useful for microbial detection from respiratory samples with high pathogen abundance [8, 10, 31]; however, basecalling accuracy and sensitivity challenges have historically limited its capacity for detecting underrepresented sequences in metagenomic datasets [32]. Targeted enrichment can overcome this through AMR signal amplification, and consistent with this, we found high concordance for AMR gene detection between nanopore and Illumina samples that underwent FLASH Cas9 targeted enrichment. Our results suggest that this method could also potentially augment AMR gene detection and resistance prediction if coupled with established nanopore mNGS workflows for detecting respiratory pathogens [8–10].

Rapid detection of AMR is essential in critically ill patients with severe bacterial infections given that time to appropriate antimicrobials correlates with mortality [33]. FLASH adds 2.5 h to standard DNA library preparation workflow and, when coupled with real-time detection of AMR genes from mNGS libraries, could potentially allow for sample to answer in under 6 h, a significant time savings compared to the ≥ 24 h required for Illumina protocols [34]. In our cohort, clinical AST required an average of 74 h, suggesting that Cas9-targeted nanopore sequencing may be a promising future approach for more rapidly identifying patients with resistant infections. We found that FLASH also enriched for AMR genes unrelated to the culture-confirmed pathogen, presumably derived from the lung microbiome. Improved methods for annotating the species-specificity of detected AMR genes may help address this issue, which otherwise could lead to increased ME due to false-positive results.

Strengths of this study include that it is the largest to assess the performance of mNGS AMR prediction in LRTI and that it systematically assessed performance for multiple classes of antimicrobials against a clinical reference standard. Further, we provide the first culture-independent assessment of healthcare exposure and resistance gene burden in the respiratory tract, and a novel demonstration of Cas9 targeted enrichment coupled with rapid nanopore sequencing. Our study also has several limitations, including sample size, spectrum of antimicrobial classes assessed, spectrum of pathogens assessed, and the need for independent validation of findings. Future work in a larger, prospective cohort with a greater diversity of bacterial pathogens and resistance mechanisms can address these limitations. In addition, a randomized clinical trial will be needed to assess the potential impact of mNGS on time to appropriate antimicrobial treatment, antimicrobial stewardship, and LRTI outcomes. Lastly, genotype to phenotype prediction remains imperfect, even for cultured isolates [7, 35]. As with other molecular testing modalities for AMR, this should be recognized when considering the utility and clinical applicability of mNGS for AMR phenotypic prediction.

## Conclusions

In summary, we characterize the utility of mNGS for predicting AMR in bacterial LRTI and demonstrate proof of concept for both epidemiological AMR surveillance and rapid resistance gene detection using Cas9 and nanopore sequencing.

## Supplementary Information

**Additional file 1: Table S1.** Clinical and demographic features of cohort.

**Additional file 2: Table S2.** mNGS detection of respiratory pathogens compared to a reference standard of clinical microbiological testing.

**Additional file 3: Dataset 1.** AMR gene database utilized for analyses.

**Additional file 4: Dataset 2.** AMR genes and guide RNAs for FLASH targeted mNGS. AMR genes and guide RNAs for FLASH targeted mNGS. A) AMR genes targeted by FLASH. B) AMR alleles and guide RNA targets. Legend: Bla = beta lactam; MLS = macrolide, Tmp/Sul = trimethoprim/sulfamethoxazole.

**Additional file 5: Table S3.** Time to return of clinical antimicrobial susceptibility testing results for culture-confirmed bacterial pathogens.

**Additional file 6: Table S4.** AMR genes detected by mNGS.

**Additional file 7: Table S5.** Performance of RNA-seq, DNA-seq and combined RNA-seq + DNA-seq for AMR prediction compared to clinical antimicrobial susceptibility testing.

**Additional file 8: Table S6.** Pathogen AMR genes detected by FLASH-mNGS.

**Additional file 9: Table S7.** AMR genes detected by FLASH coupled with Nanopore mNGS.

## Data Availability

Raw microbial sequences are available via NCBI BioProject PRJNA450137 [36], https://www.ncbi.nlm.nih.gov/bioproject/?term = PRJNA450137, and BioProject PRJNA635133 [37], https://www.ncbi.nlm.nih.gov/bioproject/?term = PRJNA635133. FLASHit [26] and SURPI*rt* [28] have been deposited on Github and are available for download at: https://github.com/czbiohub/flash and https://github.com/chiulab/SURPI-plus-dist, respectively.
