# Supplementary material for: Metagenomic prediction of antimicrobial resistance in critically ill patients with lower respiratory tract infections
Source: Genome Med. 2022 Jul 12;14:74. doi: 10.1186/s13073-022-01072-4 (PMC9275031; doi:10.1186/s13073-022-01072-4)
Supplement: Supplementary file 1 — Additional file 1: Table S1. Clinical and demographic features of cohort. [file 13073_2022_1072_MOESM1_ESM.docx]

**Table S1. Clinical and demographic features of cohort.**

|  | Total | LRTI-pos | LRTI-neg |
| --- | --- | --- | --- |
| Total enrolled | 88 | 70 | 18 |
| Age, average years | 61 | 60 | 63 |
| Female gender | 27 (31%) | 18 (26%) | 9 (50%) |
| Race/Ethnicity |  |  |  |
| African American | 4 (5%) | 3 (4%) | 1 (6%) |
| Asian | 23 (26%) | 18 (26%) | 5 (28%) |
| Caucasian | 49 (56%) | 40 (57%) | 9 (50%) |
| Other | 12 (14%) | 9 (13%) | 3 (17%) |
| Hispanic Ethnicity | 8 (9%) | 7 (10%) | 1 (6%) |
| Pneumonia Type | | | |
| Community Onset | 16 (18%) | 16 (23%) | - |
| Hospital Onset | 54 (62%) | 54 (62%) | - |
| Immunosuppression | 38 (43%) | 29 (41%) | 9 (50%) |
| Prior antibiotic use | 75 (85%) | 57 (81%) | 18 (100%) |
| Mortality (30 day) | 22 (25%) | 21 (30%) | 1 (6%) |
|  | | | |
